# Community involvement in obstetric emergency management in rural areas: a case of Rukungiri district, Western Uganda

**DOI:** 10.1186/1471-2393-12-20

**Published:** 2012-03-29

**Authors:** Simon Ogwang, Robinah Najjemba, Nazarius Mbona Tumwesigye, Christopher Garimoi Orach

**Affiliations:** 1 Makerere University School of Public Health, Kampala, Uganda; 2 Department of Community Health and Behavioural Sciences, Makerere University School of Public Health, Kampala, Uganda; 3 Department of Epidemiology and Biostatistics, Makerere University School of Public Health, Kampala, Uganda

**Keywords:** Obstetric emergency management, Maternal deaths, Community involvement, Uganda

## Abstract

**Background:**

Maternal mortality is a major public health problem worldwide especially in low income countries. Most causes of maternal deaths are due to direct obstetric complications. Maternal mortality ratio remains high in Rukungiri district, western Uganda estimated at 475 per 100,000 live births. The objectives were to identify types of community involvement and examine factors influencing the level of community involvement in the management of obstetric emergencies.

**Methods:**

We conducted a descriptive study during 2nd to 28th February 2009 in rural Rukungiri district, western Uganda. A total of 448 heads of households, randomly selected from 6/11 (54.5%) of sub-counties, 21/42 (50.0%) parishes and 32/212 (15.1%) villages (clusters), were interviewed. Data were analysed using STATA version 10.0.

**Results:**

Community pre-emergency support interventions available included community awareness creation (sensitization) while interventions undertaken when emergency had occurred included transportation and referring women to health facility. Community support programmes towards health care (obstetric emergencies) included establishment of community savings and credit schemes, and insurance schemes. The factors associated with community involvement in obstetric emergency management were community members being employed (AOR = 1.91, 95% CI: 1.02 - 3.54) and rating the quality of maternal health care as good (AOR = 2.22, 95% CI: 1.19 - 4.14).

**Conclusions:**

Types of community involvement in obstetric emergency management include practices and support programmes. Community involvement in obstetric emergency management is influenced by employment status and perceived quality of health care services. Policies to promote community networks and resource mobilization strategies for health care should be implemented. There is need for promotion of community support initiatives including health insurance schemes and self help associations; further community sensitization by empowered community based resource persons rather than health workers and improvement in quality of health care can contribute towards effective management of obstetric complications.

## Background

Maternal mortality remains a major public health problem worldwide especially in low income countries [1,2]. The reduction of maternal deaths by three-quarters, between 1990 and 2015, is a global concern, articulated as the fifth millennium development goal. Direct causes including haemorrhage, unsafe abortion, pregnancy induced hypertension, obstructed labour and sepsis contribute to 70% of maternal deaths, [1,2] while indirect causes include malaria, anaemia hepatitis, rheumatic heart disease or HIV and AIDS and factors related to health systems.

Uganda has high maternal mortality ratio estimated at 435/100,000 live births [3]. This is mainly attributed to direct causes of maternal deaths. Several factors including delay in seeking care, reaching health facilities, low socio-economic status, negative cultural beliefs and practices, and poor geographical access influence the high maternal mortality in the country [1,4-6].

Emergency Obstetric Care refers to a proven set of essential interventions geared towards reducing maternal deaths [4,7]. The core elements of emergency obstetric care include availability of essential drugs and supplies, patient referrals and the availability of skilled personnel to carry out effective interventions. Skilled health professionals working in favourable environment should be available and able to attend to every pregnancy and delivery [1,8].

Community involvement in health systems has been defined variably [9-13]. In this study, community involvement in obstetric emergencies' management are supportive measures/activities that influence/include making decisions towards seeking care; referring women; provision of essential physical and financial support, provision of transport and awareness creation by discussing issues on obstetric emergencies, communicating to health facilities/ambulance using available means like a telephone, bell, drum and or to community members regarding obstetric emergencies management so as to mobilize support for action.

The World Health Organization recognizes the importance of community involvement in health systems for several reasons [9,10]. First community involvement in health programs nurtures the aspect of ownership and individual self worthiness. Second, community resources can be leveraged to complement the available resources. Third, community involvement can expand the coverage of health interventions. Furthermore, community involvement promotes the identification of needs and delivery of health services including for obstetric emergencies' management for the beneficiary populations. Community involvement has been shown to enhance obstetric health outcomes [10,14] and improve utilization of emergency obstetric care, health seeking behaviour for obstetric care services and decision making process. Community involvement in health programs for obstetric emergencies strengthens health systems that promote utilization of services by women with obstetric emergencies [15].

Strengthening community involvement in health programme implementation has been identified as a key link towards improving maternal health through addressing the delays in seeking health care and reaching health facilities [1,4,16-19]. Thus community interventions including provision of men and women with information on sexuality and maternal health issues so that they may adopt and practice healthy sexual life style, empower women to recognize danger signs of obstetric complications.

Despite poor maternal health indices in low income countries, evidence indicates low community involvement in their management [6,8]. Knowledge about symptoms and or signs of obstetric emergencies [8,16,20], involvement and influence of men [15,20], lack of money and demographic characteristics [20], limited women autonomy in decision making to seek health care [8,21], level of women's education [8,17,18,22], perceptions on quality of health care [8], community support activities and programmes [6,16,23-28] are possible explanations in various settings. Successful community groups in facilitating access to emergency obstetric care have established funding schemes [6,29].

Evidence in Uganda, indicates that community organizations play an important role in health programmes [30]. Such institutions include burial groups, clans, insurance groups and *Engozi *groups (local name for community initiated and based groups for responding to a specific need of transporting the sick to a health facility using a locally made stretcher). In the *Engozi *groups for example membership of every household is compulsory. Members contribute cash for the purchase of a stretcher that is kept in the community for use in transporting the sick to a health facility [30].

Despite these, Uganda has inadequate comprehensive emergency obstetric care services at both primary and tertiary levels [31]. In response, the government of Uganda put in place a framework for accelerating the measures towards attainment of fifth millennium development goal target by 2015 [32]. Rukungiri district has limited health facilities that provide basic and comprehensive emergency obstetric care with two level IV health centres and two hospitals. Nearly, three quarters (77%) of the population lives within 5 km of a health facility [33]. The health indices in the district were poor, with doctor: population ratio of 1:18,513; nurse: population ratio of 1:895; midwife: child-bearing women ratio of 1:987 [34], while only 44.3% mothers deliver at health facilities [35]. So without information on how the community was involved in the management of obstetric emergencies including prevailing types of community involvement and factors influencing level of community involvement in the management of obstetric emergencies, the Rukungiri District Health Team could not design appropriate, effective and cost-efficient strategies for its improvement in efforts to reduce maternal morbidity and mortality. The study aim was to investigate community involvement in obstetric emergency management so as to inform strategies for improving maternal health status in the district. The objectives of this study were: to identify types of community involvement available/undertaken; and examine factors influencing level of community involvement in obstetric emergency management in the district.

## Methods

### Study setting

The study was conducted in Rukungiri District, western Uganda. Rukungiri district had a total population of 300,800 inhabitants; over 90% live in rural areas and engaged in subsistence agriculture [33]. The district was composed of two counties namely Rubabo (peri-urban) and Rujumbura (urban), 11 sub-counties, 77 parishes and 825 villages. Rukungiri District had two hospitals, two level IV health centres and 16 level III health centres and 42 level II health centres [36]. In 2008, the district had a total of 12 doctors, 117 nurses/midwives and 137 health staff of other categories providing health services. The district was selected based on its rural location far away (about 400 km) from Kampala capital city of Uganda, population distribution mainly in rural areas and relies on subsistence agriculture, poor maternal health status as indicated by a high maternal mortality ratio, low base of skilled health worker availability and poor nature of road network which is typical of most disadvantaged Ugandan districts.

### Study design, participants and sampling procedure

This was a descriptive study. Participants were heads of households who had lived in the district for at least two years and had children born within 24 months prior to the study. Both Rubabo and Rujumbura Counties were included in the study. The sample size was estimated using the modified Kish Leslie (1965) sampling formula with a 95% confidence interval, a precision of 5%, an estimated proportion of heads of households who make decisions to seek care for obstetric complications of 50% and a non-response rate of 10%. The sample size was calculated to be 428 heads of households but 448 were interviewed. Multistage sampling method was used to select study participants. We used simple random sampling to select six sub-counties namely Nyarushanje, Kebisoni, Rukungiri Town Council, Kagunga, Nyakagyeme and Bugangari. There were 42 parishes in the six sub-counties. From the 42 parishes, 32 villages (15.1%) were randomly selected. From each village 14 households were randomly selected, hence a total of 448 households were selected. The head of household from each selected household was interviewed.

To select the household in each village the interviewer moved to its centre. To determine the initial direction of movement a bottle was spun. The starting household was selected randomly from the number of the households in the pre-determined direction. The heads of households in the selected households were interviewed. From the first household, the subsequent nearest household was selected. However, if in any of the selected household there was no eligible respondent, the interviewer moved to the next immediate household and interviewed the head of the household, and then continued till the required number of respondents in that village (cluster) was obtained. We interviewed a total of 108 (24.1%) women and 340 (75.9%) men which corresponds to the district's sex distribution of heads of household [3].

### Data collection

Data were collected using standardized semi-structured questionnaires. The questions covered several aspects including individual characteristics, community and health services variables. Individual characteristics included sex, age, highest level of education, religion, current marital status, current employment status, tribe, number of wives/co-wives, number of pregnancies had, ever heard of a birth plan, ever/partner used a birth plan, types of messages a birth plan contained, payment for health care and saving funds to use in case of life threatening conditions during pregnancy, delivery or within 42 days after termination of pregnancy. Knowledge about symptoms of obstetric emergencies, actions to take in case obstetric emergency occurs, consequences of life threatening conditions and what communities do when obstetric emergencies develop among women in their communities were also included. Types of community involvement available/undertaken and factors influencing community involvement in the management of obstetric emergencies including health service and community factors and individual characteristics also constituted the questions.

### Quality control

The interviews were conducted in the local language Rukiga. Pre-tested semi-structured questionnaires were administered by locally-trained interviewers who were supervised by the first author. Six locally-trained interviewers (all health workers) were employed for one month. The interviewers' training was conducted for over a five days period prior to the data collection. It covered mainly objectives of the study, sampling, procedures of data collection including dos and don'ts of interviewing and getting informed consent

### Data analysis

The data were entered into EPIDATA version 3.1 and analysed using STATA version 10.0. First we carried out factor analysis to compute for the community involvement index. Prior to factor analysis the strength of inter-variable relationship including scale of reliability coefficient was assessed using Cronbach's alpha statistic test. The variable obtained after predict was changed into 3 quantiles i.e. high, medium and low.

The variables were further categorized into binary outcome (dependent variable), one value for high and the other for low community involvement. Associations between dependent variable and independent variables were tested. Independent variables were categorized into individual, community and health service factors. Individual characteristics included respondent's sex, age, employment status, whether saved money to use in the event of an obstetric emergency, perceived rating of general health facility care, rating of maternity health care, decision maker at household for health care seeking, number of wives and use of birth plans and knowledge about symptoms/signs of obstetric emergencies. Symptoms/signs of obstetric emergencies included vaginal bleeding, fits during pregnancies, high temperature within 42 days after termination of pregnancy, labour lasting more than 8 hours and swelling of the feet during pregnancy. In this study setting labour lasting more than 8 hours was considered start of prolonged labour indicating a stage when a medical doctor is expected to take action to accelerate or deliver the mother to avert adverse outcomes. Swelling of the feet though can be a normal haemodynamic physiological change, it was included to highlight the fact that not all swelling of the feet can be indicative of normal body changes but could be of progressive high blood pressure or actual severe pre-eclampsia if consistent with high blood pressure and/or protein found in urine and thus the need for women to seek health care provider's attention where the distinction is made to avoid missing a pathological state. Community factors included sensitization of the community members by their local leaders, distance to nearest health facility, distance to the nearest maternity unit (level III and IV health centres or at hospital), conditions of the roads leading to health facilities, beliefs, community actions that members undertake in the event of obstetric emergencies and availability of community social support programmes. Distance to health facility was considered a plausibly important variable. Health service factors included health education of communities about obstetric emergencies by health workers, availability of communication facility, availability of ambulance for transportation of women, perceived availability of skilled health workers at health units. Odds ratio tests were used to determine the associations between the dependent and independent variables. Multi-variable analysis was then applied to variables that were significant at P-value < 0.05 after bivariate analysis plus those with p < 0.2 but are known to be plausibly important or confounders even though they were not significant at bivariate analysis. Logistic regression was performed in order to identify independent predictors for community involvement in obstetric emergency management.

Factor analysis involving 13 variables was carried out to generate the community involvement score which was later converted into a binary variable. Four factor loadings were retained as they contributed 56.1% of the total eigen values. The community involvement index was categorized into tertiles for ease of analysis using cross-tabulations.

Analysis was carried out to identify variables that affect the level of community involvement in obstetric emergency management. Variables that were important in the model for community involvement in obstetric emergencies' management and had a p value ≤ 0.2 were included. They included saving money for use, rating of quality of general health care, rating of quality of maternal health care, sensitization of communities on signs of obstetric complications by local leaders, belief about lack of money and conducting health education by health workers. The plausibly important variables and confounders were also included: distance to nearest health facility, distance to maternity unit, sex and age even though they were not statistically significant at bivariate analysis.

### Ethical issues

Institutional consent for the study was obtained from the Makerere University School of Public Health Higher Degrees Research and Ethics Committee, Uganda National Council of Science and Technology and permission from the District Health Officer Rukungiri District. Informed verbal consent was obtained from all respondents. Research Assistants explained the purpose of the study to the respondents. Confidentiality was maintained throughout the study and thereafter. No name was indicated in the data collection tools instead unique identification numbers were used.

## Results

Most respondents 85.4% were aged 25-54 years, 95.5% were married, 81.3% had attended primary and/or secondary level, 92.9% of respondents were Christians (52.9% protestant and 40.0% catholic) while 69.5% were employed (Table 1).

**Table 1:** Socio-demographic characteristics of respondents

Characteristics	Frequency	% of 448
**Age in years**		
15-19	9	2.0
20-24	56	12.5
25-29	101	22.5
30-34	99	22.1
35-39	101	22.5
40-54	82	18.3
**Mean age (sd)**	32.5 (7.6)	
**Age range**	16 - 54	
**Marital status**		
Single	6	1.3
Married	428	95.5
Separated/divorced	7	1.6
Widow/widower	7	1.6
**Level of education**		
None	54	12.1
Primary	274	61.2
Secondary	90	20.1
Tertiary	30	6.7
**Religion**		
Protestant	237	52.9
Catholic	179	40.0
Moslem	17	3.8
Others	15	3.4
**Employment status**		
Self-employed	265	59.2
Unemployed	130	29.0
Formal employment	46	10.3
Student	7	1.6
**Tribe**		
Mukiga	222	49.6
Munyankole	124	27.7
Omuhororo	99	22.1
Others	3	0.7

Table 2 shows that the respondents were more knowledgeable about vaginal bleeding as a sign of obstetric complication compared to other signs of obstetric emergencies. Generally, females were more knowledgeable about most signs of obstetric emergencies than males. The level of knowledge about signs of obstetric emergencies significantly changed by employment status including vaginal bleeding (p < 0.01), swelling of feet (p < 0.001) and fits during pregnancy (p < 0.05).

**Table 2:** Respondents' knowledge about the signs of obstetric emergencies

Respondents' characteristics		Vaginal bleeding	Swelling of feet	Fits during pregnancy	Labour lasting more than 8 hrs	High temp within 42 days after pregnancy termination
	
	*n*	*%*	*%*	*%*	*%*	*%*
**Sex**						
Male	340	66.2	23.2	7.7	23.8	24.4
Female	108	74.1	24.1	11.1	22.2	28.7
**Age in years**						
15 - 24	65	66.2	21.5	7.7	24.6	23.1
25-29	101	66.3	21.8	9.9	17.8	29.7
30-34	99	72.7	27.3	10.1	29.3	30.3
35-39	101	73.3	28.7	9.9	20.8	20.8
40-54	82	59.8	15.9	3.7	25.6	22.0
**Marital status**						
Married	428	67.8	23.4	8.9	23.6	25.9
Single/separated/widow(er)	20	75.0	25.0	0.0	20.0	15.0
**Level of education**						
None	54	59.3	11.1	0.0	24.1	16.7
Primary	274	67.2	24.5	10.6	24.1	27.7
Secondary	90	72.2	24.4	6.7	22.2	25.6
Tertiary	30	80.0	33.3	10.0	20.0	20.0
**Religion**						
Catholic	179	70.4	27.9	10.6	24.0	25.1
Protestant	237	64.6	19.0	6.3	21.1	23.6
Moslem	17	88.2	29.4	11.8	35.3	35.3
Others	15	73.3	33.3	13.3	40.0	46.7
**Employment status‡**						
Unemployed/Student	137	**56.9**	**30.0**	**8.5**	28.5	24.6
Formal employment	46	**84.8**	**41.3**	**19.6**	26.1	17.4
Self-employed	265	**70.9**	**17.0**	**6.8**	20.8	27.6
**Tribe**						
Mukiga	222	72.1	26.1	10.4	25.7	26.0
Munyankole	124	63.7	19.4	6.5	23.4	27.4
Omuhororo/other	102	64.7	22.6	6.9	18.6	19.6
**Number of wives**						
One	303	66.3	23.1	8.6	22.8	24.8
More than one	37	64.9	24.3	0.0	32.4	21.6
**Number of pregnancies**						
One	22	72.7	31.8	4.6	31.8	22.7
Two to Four	58	75.9	24.1	10.3	24.1	29.3
≥ Five	28	71.4	17.9	17.9	10.7	32.1

**Key**: Significance is at p-value < 0.05;

**‡ **implies significance for vaginal bleeding p < 0.01, swelling of feet p < 0.001 and fits during pregnancy p < 0.05

The main support activities undertaken by community members before women developed obstetric emergencies were identification of transportation means and sensitization of communities (Table 3). The main activities undertaken when a woman developed an obstetric emergency were transportation of women and referring women to health facilities. There were significantly higher proportions of referral of women (p = 0.047) and use of ambulance at health facility (p = 0.037) in Rubabo County than in Rujumbura County (Table 3). The overall mean distance to the nearest maternity health facility where basic emergency obstetric care take place was 5.0 (range: 0.5-30.0) kilometres (km). However, mean distance to the nearest maternity was 4.2 (range: 0.5-22.0) km for Rujumbura County and 5.9 (range: 0.5-30.0) km for Rubabo County (Table 3).

**Table 3:** Community practices and support towards obstetric emergency management by County

Community practices	County				Significance (z- test p-value)
		
	Rujumbura		Rubabo		
		
	n = 238		n = 210		
	**%**		**%**		
		
**Interventions before women develop obstetric emergencies**					
Conducting planning for support to give women	7.1		6.7		0.843
Identification of transportation means to be used for women	19.8		25.4		0.164
Sensitization of women on those conditions	5.0		4.8		0.891
**Intervention when woman develops obstetric emergency**					
Taking her to health facility using stretcher	61.3		61.4		0.985
Taking her to the traditional healer	11.8		11.9		0.963
Referring her to health facility	32.4		41.4		0.047*
Mobilizing money to support her	14.3		18.1		0.273
Contacting the ambulance at health facility	1.7		5.2		0.030*

**Support programmes**	**Rujumbura**		**Rubabo**		**Total**
		
	**n**	**%**	**n**	**%**	

Community health insurance	7	9.3	8	10.1	15
Women development associations	11	14.7	6	7.6	17
Community zone saving and credit schemes	41	54.7	46	58.2	87
Community associations	12	16.0	10	12.7	22
Zone/Engozi/burial groups	4	5.3	9	11.4	13

**Distance**	**Rujumbura**		**Rubabo**		**Overall**

**Distance to nearest health facility**					
Mean distance in km	1.8		2.2		2.0
Standard deviation	1.0		2.4		1.8
Range for distance	0.5-10.0		0.2-30.0		0.2-30.0
**Distance to nearest maternity health facility§**					
Mean distance in km	4.2		5.9		5.0
Standard deviation	3.8		6.4		5.2
Range for distance	0.5-22.0		0.5-30.0		0.5-30.0

Key:*statistically significant at p < 0.05

**§**Maternity health facility includes a level III and IV health centres and hospital

Table 4 shows that community members were two times more likely to be highly involved in obstetric emergencies' management if they: were employed (AOR = 1.91, 95% CI: 1.02-3.54, p < 0.05), rated quality of maternal health care as good (AOR = 2.22, 95% CI: 1.19-4.14, p < 0.05) or were not health educated by health facility workers (AOR = 2.22, 95%CI: 1.18-4.19, p < 0.05). Low community involvement in obstetric emergencies' management was exhibited by 52% of community members who did not save money for use when obstetric emergencies developed (AOR = 0.48, 95%CI: 0.25-0.94, p < 0.05) and 88% of the community members if their local leaders' did not sensitize them on the signs of obstetric emergencies AOR = 0.12, 95%CI: 0.03-0.47, p < 0.01).

**Table 4:** Logistic regression analysis of factors associated with community involvement in obstetric emergency management

*Variable*	*Community involvement*		*Unadjusted OR*	*95% CI*	*Adjusted* *OR*	*95% CI*
	***High***	***Low***				
					
**Sex**						
Male	151	82	1.00			
Female	42	30	1.32	0.77 - 2.26	1.04	0.56 - 1.94
**Age in years**						
15 - 24	23	18	1.00			
25 - 54	170	94	0.71	0.36 -1.38	0.67	0.31 - 1.45
**Employment status**						
Unemployed/student	70	25	1.00			
Employed (formal/self)	123	87	1.98	1.16 - 3.37*	1.91	1.02 - 3.54*
**Saving money for use when obstetric emergency develops**						
Yes	139	95	1.00			
No	54	17	0.46	0.25 - 0.84*	0.48	0.25 - 0.94*
**Rating of quality of general health care**						
Poor/Fair	129	62	1.00			
Good	64	50	1.63	1.01 - 2.62*	0.91	0.48 - 1.71
**Rating quality of maternal health care**						
Poor/Fair	118	45	1.00			
Good	75	67	2.34	1.46 - 3.77***	2.22	1.19 - 4.14*
**Sensitizing communities on signs of obstetric emergencies by local leaders**						
Yes	3	14	1.00			
No	190	98	0.11	0.03 - 0.39**	0.12	0.03 - 0.47**
**Distance to the nearest health unit**						
≥ 5 km	3	5	1.00			
< 5 km	190	107	0.34	1.17 - 3.53	0.23	0.05 - 1.08
**Distance to the maternity unit**						
≥ 5 km	82	39	1.00			
< 5 km	111	73	1.38	0.85 - 2.24	1.72	0.97 - 3.04
**Conducting community health education by health workers**						
Yes	64	22	1.00			
No	129	90	2.03	1.17 - 3.53*	2.22	1.18 - 4.19*

**Key**: significance is at p < 0.05:

Whereby we get * p < 0.05; ** p < 0.01; *** p < 0.001

## Discussion

Our study showed that communities carry out several activities prior to and when women develop obstetric complications. The community interventions undertaken prior to women developing obstetric emergencies included planning for support to give women, sensitizing communities and identifying transportation means to use. This is similar to the findings in a study by Hossain and Ross which reported awareness creation on danger signs and or symptoms of obstetric complications and support for transportation of women who develop obstetric complications as key community support system interventions undertaken [37]. However, our study showed a higher level of community involvement in conducting planning for support to give women and identification of transportation means in Rubabo than in Rujumbura County. This is despite the fact that the mean distance to any nearest health facility or maternity unit is longer in Rubabo than in Rujumbura County. This can be attributed to level of need of these activities in Rubabo County.

The study revealed that the activities carried out after an obstetric emergency has developed include referral of women, using a stretcher to transport women to health facility, contacting an ambulance, mobilizing money to support women and taking women to traditional healers and were all more performed in Rubabo than Rujumbura County. However, these activities were performed more in communities least served by health services. This can partly be due to level of need of those activities in Rubabo County. We can also attribute this active community involvement to more effective community leaders/networks/groups in Rubabo County whose members conduct community mobilization or health education as part of the support activities linked to community health insurance scheme. In the study area there are community managed health insurance schemes for those members who voluntarily join. It may also be a recognition by the community of the lack of effective health services and hence the need to help themselves out in the face of adversaries or emergencies (ill health). Effort by government to support community initiatives such as through insurance schemes is very much a step in the right direction and should be scaled up.

Our study showed that several factors affect level of community involvement in obstetric emergency management including employment status and the perception of quality of maternal health care, offered by the health facilities. The finding that community members who are employed are twice more likely to be involved in obstetric emergency management may be explained by the fact that the employed members of community have the financial resources to pay for health care services and related costs. The cost of health care is a known hindrance to seeking care by women [38]. Studies in West Nile region of Uganda found financial limitations to maternal health care to be important [39]. This is expected as women would not be able to meet costs involved in accessing obstetric emergencies' services including transportation and purchase of medications. However, it is noteworthy that the only two hospitals and some lower level health facilities in the district are owned and governed as religious institutes (private not-for profit health facilities/non-governmental organisations [NGO]). These NGO partners in health to the district also support community managed insurance schemes linked to the two hospitals in Rubabo and Rujumbura Counties.

On the other hand, the finding that perceived high quality maternal health care by community members promotes high community involvement in obstetric emergency management is not surprising. This is consistent with results of a study done elsewhere in Uganda indicating that quality of health care affects its use [40]. Similarly other studies have shown that good quality of health care for obstetric emergencies can contribute to reduction of maternal mortality [5,41]. The need for good quality of health care reported in this study is consistent with findings of other studies [37,42].

We found that lack of community sensitization by local leaders on signs and symptoms of obstetric complications was negatively associated with high community involvement in obstetric emergencies' management. This is an important finding that implies the need for community mobilization for health care for obstetric emergencies by local leaders. Similar efforts by communities have been demonstrated to work in other settings [6,37]. Community mobilization is critical for planning and supporting women who develop obstetric complications. It helps in reducing delay in decision making to seek and reach health facility.

Surprisingly, our study found that when health education of community members on obstetric emergencies is done by health workers, there would be low involvement in obstetric emergencies' management. As noted above, the community members in this setting seem to respond positively to their local leaders. This may be due to community being used to taking proactive initiatives that are beneficial to them. In fact the high number of available social support programs for women who develop obstetric emergencies in this community may explain this. For example there are established community groups that use a locally made stretcher to carry women to health facilities who develop obstetric emergencies. This finding could also suggest that the health workers are actually not conducting health education [37] or if they do it does not influence knowledge and thus the desired behaviour change among community members. A study in Bangladesh, lends support to our study findings, in that, the behaviour change messages that were provided through government health interventions meant to improve knowledge and hence preparedness of community members about signs of obstetric emergencies never yielded the desired change [37]. A re-design of the communication messages with inherent use of several media and involving community members in the dissemination through community support mechanisms led to improved changes in behaviour and knowledge about signs and symptoms of obstetric emergencies. Thus community networks/groups in this study setting highlight the importance of communities in providing effective, acceptable and collective contribution to obstetric emergencies' management. It is also true that community networks can influence the individual behaviour towards seeking care for obstetric emergencies. Targeted interventions addressing community key networks and supporting resource saving/mobilization initiatives that are beneficial to the community for health care is fundamental in contributing to improved community involvement in obstetric emergencies' management. However, in the study setting, health workers conduct health education mainly at health facilities to mothers who visit antenatal clinics. The main messages delivered include general danger signs that can occur during pregnancy, nutrition during pregnancy, importance of antenatal care, services available and importance of health facility delivery. Messages with little focus on symptoms and signs of obstetric emergencies as an important subject were frequently delivered. This may also explain why there is low community involvement in obstetric emergencies management when health educated by health workers.

Our study had limitations. Taking only heads of households who had children within the last 24 months prior to the study could have excluded those who had a child but died or an abortion/foetal loss. However, these were considered and households were randomly selected into the study. The sampling of households was done by spin-the-bottle and proximity method. This has implications of less cost-effectiveness, time-consuming, and bias in the selection of households towards the middle of the village and probably those with similar socio-economic status. Elsewhere it is implied that it is not a true probability-based sampling method due to the higher probability for selection of the centrally-located households [43]. However, this holds true if the households in the centre of the village and those at its edge are different in terms of socio-economic status. This could not be established by this investigation. Evidence shows that using the global positioning system (GPS) to select a point on the edge of map and using the approach of selecting a reference on a generated grid map of the village are alternatives to spin-the-bottle and proximity procedure [43].

## Conclusions

Types of community involvement in this setting include community practices and support programmes for obstetric emergencies' management. Community involvement in obstetric emergencies is influenced by employment status and perceived quality of health care services. Policies to promote community networks and their resource mobilization strategies towards obstetric emergencies' management should be implemented. The findings suggest the need to empower community own resource persons to educate community members rather than by health workers, improve quality of health care and promote income gainful initiatives for community members in order to empower them to use health services that can contribute to averting maternal deaths.

## Competing interests

The authors declare that they have no competing interests.

## Authors' contributions

SO initiated the concept for the study, participated in designing the study, supervised fieldwork and data collection, performed data analysis and interpretation and drafted the manuscript and coordinated its writing. RN participated in designing the study. NMT participated in designing the study, data analysis and interpretation. CGO participated in designing the study, data analysis and interpretation. All authors participated in revising and approved the final manuscript.

## Authors' information

Simon Ogwang MB ChB, MSc, MPH, Makerere University School of Public Health, Kampala, Uganda

Robinah Najjemba MB ChB, MPH. Department of Community Health and Behavioural Sciences, Makerere University School of Public Health, Kampala, Uganda.

Nazarius Mbona Tumwesigye Bstat, MA Demo, MSc, PhD Senior Lecturer at the Department of Epidemiology and Biostatistics, Makerere University, Kampala, Uganda.

Christopher Garimoi Orach, MB ChB, DPH, MMed, MPH, PhD is Senior Lecturer at the Department of Community Health and Behavioural Sciences, Makerere University School of Public Health, Kampala, Uganda.

## Pre-publication history

The pre-publication history for this paper can be accessed here:

http://www.biomedcentral.com/1471-2393/12/20/prepub
